# Administration of adipose tissue-derived mesenchymal stem cells and bone marrow-derived mesenchymal stem cells in anosmic mice

**DOI:** 10.55730/1300-0144.6028

**Published:** 2025-04-05

**Authors:** Burak HAZIR, Alper CEYLAN, Emin Ümit BAĞRIAÇIK, Duygu DAYANIR, Betül ÖĞÜT, Nihan ÖRÜKLÜ, Mücahit YALÇIN, Berkay ŞİMŞEK, Muammer Melih ŞAHİN

**Affiliations:** 1 Department of Otorhinolaryngology, Ankara City Hospital, Ankara, Turkiye; 2 Department of Otorhinolaryngology, Faculty of Medicine, Gazi University, Ankara, Turkiye; 3 Department of Immunology, Faculty of Medicine, Gazi University, Ankara, Turkiye; 4 Department of Histology and Embryology, Faculty of Medicine, Gazi University, Ankara, Turkiye; 5 Department of Pathology, Faculty of Medicine, Gazi University, Ankara, Turkiye; 6 Department of Otorhinolaryngology, Bartın State Hospital, Bartın, Turkiye

**Keywords:** Smell, anosmia, olfaction disorders, stem cells, mesenchymal stem cells

## Abstract

**Background/aim:**

Anosmia, a serious condition that affects the sense of smell, has no universally agreed-upon treatment. Adult stem cells are considered a potential option for treating anosmia. This study aims to evaluate the efficacy of mesenchymal stem cells derived from different tissues in an anosmic mouse model induced by 3-methylindole (3-MI).

**Materials and methods:**

In our study, 36 mice with 3-MI-induced anosmia were divided into subgroups. Anosmia was confirmed by performing a food-finding test (FFT) in each group. Intranasal phosphate-buffered saline was administered to the first group, adipose tissue-derived mesenchymal stem cells (ADSCs) to the second group, and bone marrow-derived mesenchymal stem cells (BMSCs) to the third group. Stem cells were obtained from green fluorescent protein (GFP)-transgenic mice. Olfactory function was evaluated weekly using the FFTs. Mice were sacrificed at the second and fourth weeks following 3-MI injection and examined histopathologically.

**Results:**

Compared to the control group, stem cell-transplanted groups demonstrated significantly improved food-finding times measured at week 2 and week 4 FFTs following the 3-MI injection (respectively; p = 0.001, p = 0.008). Additionally, increased olfactory marker protein expression and olfactory epithelial thickness, along with reduced epithelial damage, were observed in the stem cell-transplanted groups compared to the control group (p < 0.05). Histologically, BMSCs showed greater efficacy than ADSCs in promoting olfactory regeneration. Furthermore, GFP+ cells were detected in the olfactory epithelium and olfactory bulbs of the stem cell-transplanted groups.

**Conclusion:**

It was observed that intranasally transplanted stem cells could reach the damaged olfactory region and enhance olfactory regeneration and functional recovery. Both ADSCs and BMSCs were effective in treatment and appear to be promising therapeutic modalities.

## 1. Introduction

Better understood in its absence, smell is one of the five senses that has unique characteristics. Although it varies according to age groups, it is estimated that approximately 20% of the population has hyposmia and 5% has functional anosmia [1, 2]. The sense of smell is perceived as a result of the stimulation of olfactory receptor neurons (ORN) in the olfactory neuroepithelium (ONE) by odor molecules. Formed by the axons of ORN, the olfactory nerve is the only cranial nerve that directly contacts the outside [3]. This contact can cause degeneration and may be the reason why the olfactory nerve is the only nerve capable of regeneration [4]. Because it has the ability to regenerate, it can often recover completely when damaged; however, partial or complete loss of function may still occur in some cases. Although numerous agents have been tried in treatment, there is no widely accepted option with broad effectiveness, as the pathophysiology remains unclear. Systemic and intranasal steroids, vitamin A, statins, zinc, cellular therapies, and olfactory training have been proposed in the literature as therapeutic strategies [1, 5–13].

Adult stem cells offer a promising method that has been tested in the treatment of various diseases. Replacing damaged stem cells of the degenerated ONE with new cells is considered a potential treatment for olfactory disorders. Stem cell transplantation contributes to neural regeneration by releasing or inducing neurotrophic growth factors and proangiogenic cytokines [14]. Several preclinical studies have investigated the regenerative effects of mesenchymal stem cells (MSCs) on the olfactory system [6, 12, 13, 15]. For instance, Kim et al. demonstrated that systemic administration of adipose tissue-derived mesenchymal stem cells (ADSCs) promoted olfactory epithelium regeneration [6]. Ochi et al. reported that bone marrow-derived mesenchymal stem cells (BMSCs) could migrate to the olfactory mucosa after transplantation but failed to show olfactory marker protein (OMP) expression [12]. Moreover, Jo et al. showed that transplantation of BMSCs in rats enhanced olfactory recovery by increasing NGF and BDNF expression, further supporting their potential in olfactory regeneration [15]. However, to our knowledge, no prior study has directly compared the therapeutic efficacy of adipose tissue-derived and bone marrow-derived mesenchymal stem cells using the same anosmia model. This study aimed to fill that gap by evaluating and comparing the functional and histological outcomes of intranasally transplanted ADSCs and BMSCs in a 3-methylindole-induced anosmic mouse model.

## 2. Materials and methods

Our study was approved by the local ethical committee. A total of 36 healthy C57BL-6 male mice (aged 8–10 weeks and weighing 18–22 g) whose olfactory functions had been found to be intact through a food-finding test (FFT) were included. Mice were randomly divided into three groups, each containing 12 mice. The first group received phosphate-buffered saline (PBS), the second received ADSCs, and the third received BMSCs intranasally one week after the 3-methyl indole (3-MI) injection. A total of five transgenic C57BL/6-Tg (CAG-EGFP)1Osb/J mice (Jackson Laboratory, Bar Harbor, USA) were included to prepare stem cells. A dose of 300 μg/g 3-MI (Sigma-Aldrich, St. Louis, USA) was administered intraperitoneally to all the groups to induce anosmia. Odor functions were evaluated through weekly FFTs. Six mice were randomly selected from each group and sacrificed in the second week; the remaining mice were sacrificed in the fourth week following the 3-MI injection before histological examination was performed.

### 2.1. Food-finding test

FFT was designed by reviewing existing models used in previous studies [7, 10, 16]. Animals were given one piece of cheese from the same brand daily to become accustomed to its taste and smell. They fasted for 24 h and were allowed only water. A T-shaped maze was designed for the test. A piece of cheese (2 × 1 cm) was placed at one end of the maze, whose bottom was covered with sawdust similar to that was used in the cages. All the tests were recorded with a video camera. Each mouse was tested five times during an FFT. Mice that could not find the cheese within 3 min were considered “failed”. The test time of the failed mice was recorded as 180 s. Mice that could not find the cheese within 3 min in three out of five tests were considered “unsuccessful”. The minimum and maximum times were excluded, and the average of the remaining three tests was calculated. The first test was performed to evaluate olfactory functions before the 3-MI injection and the second one week after the 3-MI injection to show anosmia. Then, the remaining tests were conducted weekly (FFT 1, FFT 2 and FFT 3).

### 2.2. Preparation of stem cells

Stem cells were prepared from green fluorescent protein (GFP)-transgenic C57BL6 mice. Based on criteria published by the International Cell Therapy Society for the characterization of mesenchymal stem cells, they were produced through a standardized method whose characterization and capacity of differentiation had been exhibited by stem cells produced previously in our stem cell center [17–19].

To prepare ADSCs, inguinal adipose tissue was isolated and cut into small pieces. Then it was mechanically lysed in a buffer solution containing type I collagenase enzyme (Sigma-Aldrich, St. Louis, USA) and incubated at 37 °C for 30 min. After incubation, it was passed through a cell filter, washed by centrifugation, and inoculated into sterile cell culture flasks (Greiner Bio-one, Kremsmunster, Austria). Dulbecco’s Modified Eagle Medium (DMEM) (Sigma-Aldrich, St. Louis, USA) containing penicillin/streptomycin, L-glutamine (Sigma-Aldrich, St. Louis, USA) and 10% fetal bovine serum (FBS) was used as medium. Cells were incubated at 37 °C in a 5% CO2 incubator (Sanko, Osaka, Japan). The medium was changed every three days during incubation. Once existing flasks had sufficient filling, the adherent cells were transferred to new flasks.

To prepare BMSCs, the femur and tibia bones of mice were used. Once the epiphyses of the bones were separated, the bone marrow was aspirated by washing with DMEM-containing antibiotics. After passing through the cell filter, the bone marrow aspirates were washed by centrifugation and incubated at 37 °C in an environment containing 5% CO2. DMEM containing penicillin/streptomycin, L-glutamine, and 10% FBS was used as medium. During incubation, the medium was refreshed every three days and the cells were transferred as they proliferated. The MSCs transferred to anosmic mice were obtained from the third passage of cultures derived from both bone marrow and adipose tissue.

Isolated and purified BMSCs and ADSCs were characterized by immunophenotyping using flow cytometry. All the antibodies were purchased from the same company (Sony Biotechnology, California, USA). Cells with negative CD45 markers and positive CD44, CD90.2, CD29, and CD73 markers were accepted as MSCs. Before characterization, the stem cells were examined under an immunofluorescence microscope to assess their GFP intensities.

### 2.3. Administration of stem cells

1 × 10^7^ cells suspended in 50 μL of PBS were administered to the mice that exhibited anosmia in the week 1 FFT following 3-MI injection. Mice were mildly anesthetized during administration. Since it has been shown in the literature to be a safe technique, stem cells were transplanted into each nostril using a micropipette in a volume of 25 μL, administered as 5-μL drops at 5-min intervals [11, 20]. Similarly, the same volume of PBS was administered intranasally to the control group under mild anesthesia.

### 2.4. Histological examination

Animals were sacrificed with high-dose anesthesia and then decapitated. Next, for each mouse, the lower jaw was separated and the head was divided into two sides in the sagittal plane after the surrounding muscles were removed. One side was placed in formaldehyde for histological examinations while the other side was isolated and placed in a separate container to investigate transplanted GFP+ stem cells. All the histological evaluations were performed by blinded investigators. In order to analyze GFP+ cells without any staining, one side of the ONE was carefully removed from the ethmoturbinate and septum using microsurgical instruments under a microscope, and the olfactory bulb (OB) was separated from the surrounding tissues and then isolated.

To inspect stem cell engraftment, 5 μm sections were taken at −20 °C with a cryostat device (Leica CM1900, Germany). With the help of an inverted immunofluorescent microscope, blinded investigators analyzed the sections and assessed GFP+ cells.

For histomorphological examination, the samples were first fixed in a 10% neutral formaldehyde solution for 72 h and then transferred to the decalcification solution. After washing all the tissues, they were passed through increasing alcohol series (50%, 70%, 80%, 90%, and 100%) and xylol. Next, they were embedded in paraffin, thereby generating paraffin blocks. Sections of 4–5 micron thickness were taken from the emergent paraffin blocks and stained with hematoxylin-eosin (HE). Histomorphological changes and olfactory marker protein uptake in the obtained samples were examined with a light microscope. Loss of cilia, vacuolization, and inflammatory cell infiltration were scored to evaluate epithelial damage (0: no damage, 1: mild, 2: moderate, 3: severe with a total maximum score of 9). Epithelial thickness was measured from the same three regions (ethmoturbinate, two peripheral, and one central), and the resulting figures were averaged [21].

For immunohistochemical examinations, the samples were first kept in xylol twice for 15 min each time. After extraction from xylol, they were kept in ethyl alcohol solutions at 100%, 96%, 90%, 80%, and 70% concentrations, respectively, for 10 min and then held in distilled water twice for 5 min each time. Afterwards, the tissues lined up on the immunohistochemistry bar in a humid environment were scratched with PAP-Pen and washed with PBS three times for 3 min each. Samples treated with serum blocking solution (Genemed Biotechnologies, USA) for 10 min were incubated overnight at +4 °C with primary antibodies of OMP and CD45. After incubation, a 3% hydrogen peroxide solution was applied for 15 min to the samples washed with PBS. Next, a secondary antibody with biotin (Genemed Biotechnologies, USA) was applied and the samples were washed again three times with PBS for 3 min each. After the application of chromogen containing diaminobenzidine (DAB) substrate (Thermo Fisher Scientific, MA, USA), the samples were observed until a visible immune reaction occurred. Mayer’s hematoxylin was used as a background dye for the samples washed with PBS. OMP uptake was assessed in cell counts from seven independent fields selected for each slide.

### 2.5. Statistical analysis

The SPSS 26.0 program (SPSS Inc., Chicago, IL) was used for statistical analysis. Frequency and percentage were provided for categorical variables, while mean, median, standard deviation, minimum, and maximum values were given for numerical variables. For independent groups, quantitative data were compared using the Mann-Whitney U and Kruskal-Wallis tests, and qualitative data using the chi-square test, Fisher’s exact test, or Fisher-Freeman-Halton exact test. For comparisons involving more than two independent groups, if a statistically significant difference was detected, pairwise comparisons were subsequently performed using the Mann-Whitney U test with Bonferroni correction. A significance threshold of p < 0.016 was considered statistically significant in these pairwise comparisons. For dependent groups, the Friedman test or the Wilcoxon test was used. A p-value less than 0.05 was considered statistically significant.

## 3. Results

### 3.1. Food-finding test

**Figure 1:**
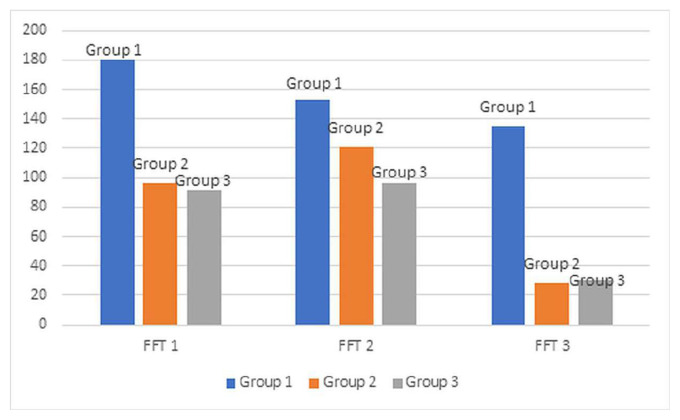
Average food-finding times *FFT: Food-finding test

Averages of food-finding times and success status of mice in groups are shown in Table 1 and Figure 1. FFT values revealed that anosmia developed in all the mice after 3-MI injection. While 100% of the mice in Group 1 were unsuccessful in FFT 1, the rates were 25% and 33% in Groups 2 and 3, respectively.

The difference in average times between the groups in the FFT 1 (p = 0.001) and in the FFT 3 (p = 0.008) was found to be statistically significant. However, no statistically significant difference was found between the groups in FFT 2 (p = 0.276). While there was a statistically significant difference between Group 1 and both Group 2 and Group 3 in the FFT 1 and 3, there was no statistically significant difference between Group 2 and Group 3.

### 3.2. Evaluation of transplanted stem cells

**Figure 2:**
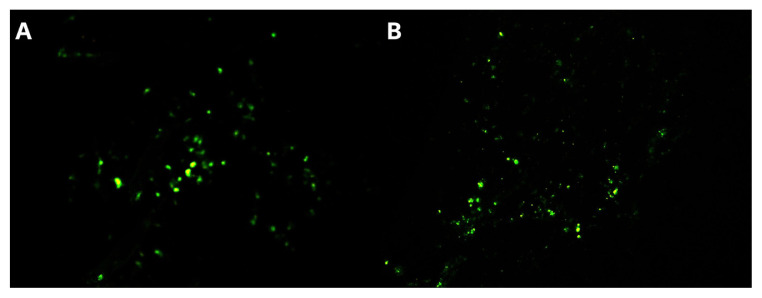
Second week images of GFP+ BMSCs (10x). **A.** Olfactory Epithelium, **B.** Olfactory Bulb.

Before transplantation, all stem cells were confirmed to express GFP under an immunofluorescence microscope. Microscopic analysis of OB and ONE isolated from all the stem cell-transplanted mice demonstrated the presence of GFP+ cells. Findings suggest that intranasally administered BMSCs and ADSCs settle in ONE and OB and then proliferate in these regions over time. Figures 2A and 2B show GFP+ cells in tissues.

### 3.3. Histomorphological findings

Inflammatory cell infiltration of the olfactory epithelium, loss of cilia, and vacuolization status were scored in the histomorphological evaluation. The results obtained in the second and fourth weeks are shown in Tables 2 and 3. In the analysis performed to compare the total epithelial damage scores between the groups, statistically significant differences were observed among all groups at both time points (p < 0.05).

When the epithelial statuses of Group 1 in the second and fourth weeks were compared, there were signs of improvement in the condition of the cilia and vacuolization, but no statistically significant difference was observed (p = 0.317). Though there was an improvement in the total epithelial damage score of Group 2 in the second and fourth weeks, it was not statistically significant (p = 0.063). The total damage score of Group 3 showed a statistically significant decline in the fourth week compared to the second week (p = 0.034)

**Figure 3:**
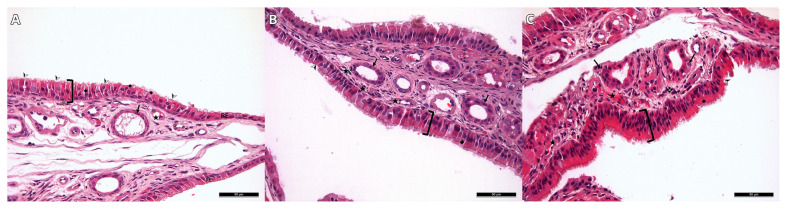
Images of the olfactory epithelium in second week (Hematoxylin-eosin). **A.** Group 1, **B.** Group 2, **C.** Group 3. **]**: Epithelial thickness Black arrow: Duct of the gland ♦: Vacuolization ❖: Inflammatory cell infiltration ★: Vascularization ➢: Loss of cilia.

There was a statistically significant difference between the groups in terms of ONE thickness. Group 3 had the thickest ONE whereas Group 1 had the thinnest (Table 4). A statistically significant difference was found between all the groups in the pairwise comparisons (p < 0.016). When the second- and fourth-week samples of a group were compared, it was seen that epithelial thickness was found to increase in all groups, which was also statistically significant in all the groups (p < 0.05) (Figures 3A–3C).

### 3.4. Immunohistochemical findings

Analysis showed that there were significant differences between the groups in terms of the number of OMP+ cells in the second and fourth weeks (Table 5).

**Figure 4:**
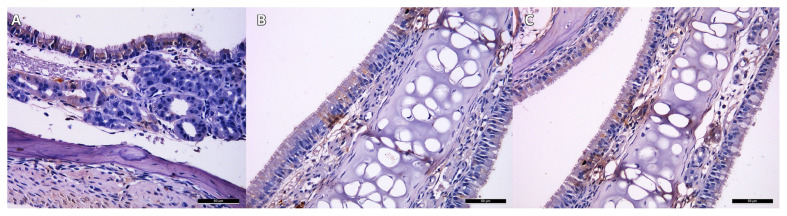
Images of the OMP + cells in second week. **A.** Group 1, **B.** Group 2, **C.** Group 3.

The number of OMP+ cells was found to be the lowest in the control group and the highest in Group 3 in both weeks. Pairwise comparisons of the groups demonstrated that the differences between all the groups in all the time periods were found to be statistically significant (p < 0.016) (Figures 4A–4C).

**Figure 5:**
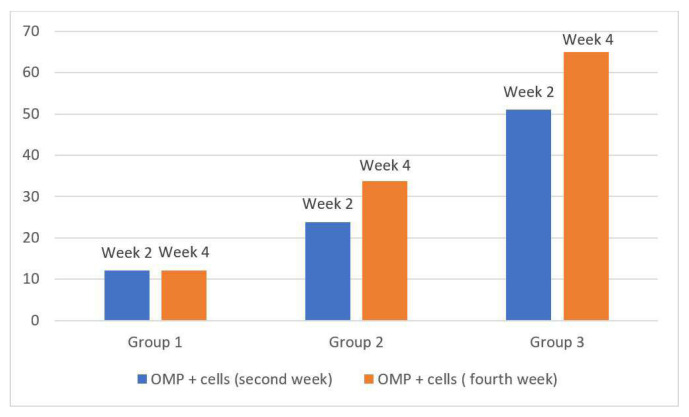
Number of cells expressing olfactory marker protein. *OMP: Olfactory marker protein

Considering OMP+ cell changes encountered within the groups in the second and fourth weeks, while there was no significant change in Group 1, OMP+ cells exhibited a statistically significant increase in Groups 2 and 3 (p < 0.05, Figure 5).

## 4. Discussion

In this study, we investigated the effects of intranasally transplanted ADSCs and BMSCs on olfactory regeneration in an anosmic mouse model. Our findings showed that both ADSCs and BMSCs significantly improved olfactory function as demonstrated by FFTs, increased olfactory epithelial thickness, and higher OMP expression compared to controls. Among the stem cell groups, BMSCs were associated with more prominent histological and functional recovery. These results suggest that mesenchymal stem cells, particularly BMSCs, hold therapeutic potential for the treatment of olfactory disorders.

Widely studied in the literature, ADSCs and BMSCs are capable of osteogenic, chondrogenic, and adipogenic differentiation, and are arguably able to differentiate into neuronal or cardiogenic structures under appropriate environmental conditions [13, 22–24]. The possibility of differentiation into these neural structures has brought up the use of MSCs in neural degeneration. Apart from their differentiation potential, MSCs can also function by secreting neurotrophic factors and angiogenic cytokines [25,26]. Nerve growth factor (NGF) and brain-derived neurotrophic factor (BDNF) are some examples of the cytokines secreted by MSCs thought to improve olfactory functions [15]. Jo et al. showed an elevation of NGF and BDNF expression and Kwon showed an elevation of NGF expression in their studies [15, 27]. Therefore, the use of stem cell therapies to induce regeneration is thought to be effective in olfactory disorders as well as in other neurodegenerative diseases.

Studies on neural stem cells have demonstrated their therapeutic efficacy [11, 20]. However, in clinical practice, it is difficult to access and isolate them. Thus, MSCs, proven to be similarly effective in animal studies, provide ease of use [6, 15, 27–28]. Although ADSCs and BMSCs are both MSCs, they are derived from different tissues and may vary in cell yield, differentiation capacity, and cytokine/chemokine secretion profiles [29–31]. ADSCs are easier and less invasive to obtain compared to BMSCs. In addition, it is possible to obtain and produce ADSCs in large quantities and with repetitive applications. Our study also aims to evaluate these differences.

There are studies in the literature comparing ADSCs and BMSCS. For instance, Karaoz et al. reported that the immunosuppressive effect of BMSCs is stronger than that of other stem cells [32]. Takahashi et al. reported that ADSCs are resistant to stress and hypoxia, and therefore, their effect on healing may be greater than that of BMSCs, as they persist in greater numbers within the tissue [33]. However, BMSCs and ADSCs have been shown to have similar efficacy in the treatment of some conditions such as spinal cord injury and neuropathic pain [33–35].

A large number of behavioral tests have been described in the literature to evaluate olfactory functions in animals. There are two types of behavioral tests: avoidance and preference [36]. It is also possible to assess olfactory functions in animals through an electroolfactogram; however, a behavioral test was chosen for its simplicity, reproducibility, and demonstrated reliability in assessing olfactory function. Considering success status and average food-finding times in FFTs 1 and 3, there was a significant difference between the control group and stem cells-transplanted groups. This suggests that stem cells improve olfactory function during the first week of therapy.

Yasak et al. analyzed olfactory epithelial degeneration by scoring inflammatory cell infiltration, epithelial cell vacuolization, and cilia damage in the olfactory epithelium [21]. A similar scoring technique was used in our study. Besides, olfactory epithelial thickness was measured and compared between groups. Results obtained in the second and fourth weeks indicated that Groups 2 and 3 had thicker epithelium. Likewise, degeneration scores of the stem cell-transplanted groups were lower than those of the control group. Comparisons between stem cell-transplanted groups showed that BMSCs-transplanted mice had higher epithelial thickness and lower epithelial damage scores.

Present in mature olfactory neurons, OMP is a protein used in the literature to evaluate recovery in anosmia-induced models [7]. While Ochi et al. reported that BMSC-transplanted mice did not express OMP, Lee et al. showed that the neural stem cell transplanted group expressed OMP in their study [12, 20]. OMP+ cells were also analyzed in our study, and it was observed that the number of OMP+ cells in the second and fourth weeks was significantly higher in the stem cell-transplanted mice compared to those in the control group. Among the stem cell groups, the number of OMP+ cells in the BMSCs-transplanted group was higher than that in the ADSCs-transplanted group. Histological findings suggested that the BMSC group had better improvement in the subjects than the ADSCs group, however, when compared to the control group, both MSCs had a positive impact on recovery.

There are preclinical studies in the literature showing that stem cell treatments are successful in olfactory degeneration. However, our study investigated the efficacy of intranasal transplanted ADSCs in an anosmia-induced mouse model and also compared the efficacy of ADSCs with that of BMSCs. There is no study making this comparison. Behavioral test results indicated faster functional recovery in stem cell-treated mice. Stem cell applications offered quicker improvement histologically in terms of olfactory epithelial regeneration. BMSCs seemed to be more effective than ADSCs in olfactory degeneration.

Since this was an animal study, it is possible that our results may differ in humans. Rodents are more odor-dependent than humans, and their ORN turnover is faster. Although chemically induced anosmia produces robust degeneration, it cannot simulate a chronic disease model similar to that encountered in humans. Furthermore, our study evaluated the engraftment of stem cells only under an immunofluorescent microscope. Immunohistochemical and genetic methods are likely to produce objective results for engraftment. Lastly, analyzing the proteins expressed by the transplanted cells may help determine their differentiation status.

Stem cell applications have the potential to be a new treatment option for olfactory disorders. However, there is a need for more objective studies. We think that our study will guide future clinical and preclinical studies.

## Figures and Tables

**Table 1:** Success status of the groups in food-finding test.

		Group 1		Group 2		Group 3		P-value
		n	%	n	%	n	%	
FFT 1*	Unsuccessful	12	100	3	25	4	33.3	<0.001
	Successful	0	0	9	75	8	66.7	
FFT 2**	Unsuccessful	4	66.7	2	33.3	2	33.3	0.589
	Successful	2	33.3	4	66.7	4	66.7	
FFT 3***	Unsuccessful	4	66.7	0	0	0	0	0.015
	Successful	2	33.3	6	100	6	100	

* Two weeks after 3-MI injection

** Three weeks after 3-MI injection

*** Four weeks after 3-MI injection

**Table 2:** Histopathological scores of the second week.

**Histopathological scores**		**Scores**	**Group 1**	**Group 2**	**Group 3**	**P value**
	**Infiltration**	**0**	0	0	0	=0.002*
		**1**	0	0	5	
		**2**	3	5	1	
		***3***	*3*	1	0	
	**Vacuolization**	**0**	0	0	0	<0.001*
		**1**	0	3	5	
		**2**	0	3	1	
		**3**	6	0	0	
	**Cilia damage**	**0**	0	0	0	<0.001*
		**1**	0	4	4	
		**2**	0	2	2	
		**3**	6	0	0	
	**Total score**		**Average ± SD**	**Average ± SD**	**Average ± SD**	=0.001**
			8.5 ± 0.54	5 *±* 0.89	3.67 *±* 0.51	

* Fisher-Freeman-Halton-Exact

** Kruskal Wallis

**Table 3:** Histopathological scores of the fourth week.

**Histopathological scores**		**Scores**	**Group 1**	**Group 2**	**Group 3**	**P value**
	**Infiltration**	**0**	0	0	0	<0.001*
		**1**	0	4	6	
		**2**	0	2	0	
		***3***	6	0	0	
	**Vacuolization**	**0**	0	0	0	<0.001*
		**1**	0	3	6	
		**2**	1	3	0	
		**3**	5	0	0	
	**Cilia damage**	**0**	0	0	2	=0.002*
		**1**	0	5	4	
		**2**	4	1	0	
		**3**	2	0	0	
	**Total score**		**Average ± SD**	**Average ± SD**	**Average ± SD**	<0.001**
			8.17 *±* 0.41	4 ± 0	2.67 ± 0.52	

* Fisher-Freeman-Halton-Exact

** Kruskal Wallis

**Table 4:** Olfactory epithelial thicknesses of the groups.

	Group 1		Group 2		Group 3		P value
	Average ± SD	Median	Average ± SD	Median	Average ± SD	Median	
**Olfactory epithelial thicknesses (μm) (2nd week)**	27.32 *±* 0.96	27.35	47.68 *±* 0.28	47.55	68.99 ± 0.81	69.07	<0.001*
**Olfactory epithelial thicknesses (μm) (4th week)**	38.91 ± 1.06	39.1	54.55 ± 0.63	54.85	80.35 ± 0.2	80.32	<0.001*

* Kruskal Wallis

**Table 5:** Number of OMP+ cells of the groups.

	Group 1		Group 2		Group 3		P value
	Average ± SD	Median	Average ± SD	Median	Average ± SD	Median	
**Number of OMP+cells (2nd week)**	12.05 ± 0.93	12.5	23.88 ± 3.03	22.83	51.05 ± 3.39	51.67	<0.001*
**Number of OMP+cells (4th week)**	12.05 ± 0.80	11.83	33.83 ± 2.09	34	64.94 ± 3.25	64.83	=0.001*

* Kruskal Wallis
